# Exploring the Use of Pictograms in Privacy Agreements to Facilitate Communication Between Users and Data Collecting Entities: Randomized Controlled Trial

**DOI:** 10.2196/34855

**Published:** 2023-01-25

**Authors:** Larissa Ugaya Mazza, Laura Xavier Fadrique, Amethyst Kuang, Tania Donovska, Hélène Vaillancourt, Jennifer Teague, Victoria A Hailey, Stephen Michell, Plinio Pelegrini Morita

**Affiliations:** 1 School of Public Health Sciences Faculty of Health University of Waterloo Waterloo, ON Canada; 2 Institute of Health Policy, Managment, and Evaluation University of Toronto Toronto, ON Canada; 3 Canadian Standards Association Group Montreal, QC Canada; 4 Victoria Hailey Group Corporation Toronto, ON Canada; 5 Canadian Standards Association Group Etobicoke, ON Canada; 6 eHealth Innovation Techna Institute University Health Network Toronto, ON Canada; 7 Department of Systems Design Engineering University of Waterloo Waterloo, ON Canada

**Keywords:** pictograms, privacy agreements, user trust, transparency

## Abstract

**Background:**

Privacy agreements can foster trust between users and data collecting entities by reducing the fear of data sharing. Users typically identify concerns with their data privacy settings, but due to the complexity and length of privacy agreements, users opt to quickly consent and agree to the terms without fully understanding them.

**Objective:**

This study explores the use of pictograms as potential elements to assist in improving the transparency and explanation of privacy agreements.

**Methods:**

During the development of the pictograms, the Double Diamond design process was applied for 3 instances of user interactions and 3 iterations of pictograms. The testing was done by performing a comparative study between a control group, which received no pictograms, and an experimental group, which received pictograms. The pictograms were individually tested to assess their efficacy by using an estimated comprehension of information symbols test.

**Results:**

A total of 57 participants were recruited for the pictogram evaluation phase. With the addition of pictograms, the overall understanding improved by 13% (*P*=.001), and the average time spent answering the questions decreased by 57.33 seconds. A 9% decrease in perceived user frustration was also reported by users, but the difference was not significant (*χ*^2^_4_=4.80; *P*=.31). Additionally, none of the pictograms passed the estimated comprehension of information symbols test, with 7 being discarded immediately and 5 requiring further testing to assess their efficacy.

**Conclusions:**

The addition of pictograms appeared to improve users’ understanding of the privacy agreements, despite the pictograms needing further changes to be more understandable. This proves that with the aid of pictographic images, it is possible to make privacy agreements more accessible, thereby allowing trust and open communication to be fostered between users and data collecting entities.

**Trial Registration:**

ClinicalTrials.gov NCT05631210; https://clinicaltrials.gov/ct2/show/NCT05631210

## Introduction

Privacy agreements fulfill the important role of helping users understand how their data will be used by data collecting entities [1]. The role of privacy agreements is to not only provide users with the chance to decide whether they want to disclose their data to an entity but also foster trust and reduce users’ concerns about data sharing [1,2].

Many users are concerned about personal data collection, and privacy agreements may alleviate these concerns. However, due to the complexity of privacy agreements, there are barriers to understanding data use [3], which result in users agreeing to terms that they do not fully comprehend [4]. This paper explores the use of pictograms as a potential way to improve the transparency of privacy agreements and users’ understanding of privacy agreements.

Most studies about pictograms used as communication tools focus on pictograms that depict pharmaceutical- and health-related information [5-19] or hazardous substances and their safe handling [7,20-31].

Pictograms are useful when communicating certain types of information for which language, literacy, and reaction times can be barriers [32]. For example, some studies have shown that pictograms are beneficial for facilitating danger recognition and the understanding of precautionary measures [5,7,8,12,13,18,22,31,33]. Some of the advantages of using pictograms instead of written words are that they can facilitate faster recognition and remembrance during a second encounter and can improve the understanding of communicated messages for people with visual deficiencies or low literacy levels and people who are unfamiliar with the language used. Chief among these advantages is that pictograms can be more easily understood than their written counterparts [8,9,18,20,26,28,30,31]. When it comes to health care, pictograms have been shown to be better at informing patients about examination preparation [34].

Nevertheless, pictograms are not the solution for all communication problems. As Spinillo [35] argues, pictograms should be used judiciously, since images are more appropriate for representing material things, relative sizes, and simultaneous concepts. However, they are often inadequate for representing general or abstract concepts [35,36].

In this paper, pictograms will be defined as *“*graphic images that immediately show the user of a hazardous product what type of hazard is present. With a quick glance, [the user] can see, for example, that the product is flammable, or if it might be a health hazard*”* [37]. Pictograms are composed of both graphic and textual parts. The graphic parts include the border and the symbol, that is, a black image inside the border [37]. The textual part comprises bolded text indicating the name of the pictogram and a legend (in brackets) with a description of the hazard.

Because images are not a global language, they cannot be used for different population groups without the risk of losing or changing their meaning [8,10,20,32,35]. This makes it important to consider the specific target group when developing pictograms and to rigorously test pictograms throughout the design process [5,6,9,10,13,19,20,28,31,35].

When considering pictograms overall, Wogalter [28] talks about the four main purposes of a warning in his book *Handbook of Warnings*. In it, he says that a warning must (1) communicate important safety information; (2) influence or modify a person’s behavior to improve their safety; (3) reduce or prevent accidents, injuries, damage, or health problems; and (4) serve as a reminder for those that are already aware of the danger.

There are 4 components in the warning context that affect a pictogram’s creation and implementation [26], as follows: (1) the source (the designer, sender, or originator of the warning message), (2) the medium (how the message is being displayed; eg, visual, auditory, etc), (3) the message (the content), and (4) the receiver (the target audience that the warning seeks to reach).

According to Laughery and Wogalter [26], for a message to flow effectively, it must go from one component to the next in a linear fashion. If the connection is severed at any point, the flow can be broken, resulting in the failure to deliver the warning.

To avoid this, the involvement of users is imperative not only for testing but also for the design process. User involvement is invaluable for the inclusion of previously overlooked elements that result in improved performance [5,6,8-10,12,14,16,19, 28,31,32].

A warning can have many different parts, and it is the role of the designer to combine them effectively [26]. Each component may serve different purposes and change when directed at different users. An example of this would be using more technical language when dealing with specialists but using simpler terminologies with novices [26].

With regard to the visual components, a semiotic study separated them into the following two categories: transparent and opaque components [20]. Transparent symbols highly resemble their real-life counterparts, have guessable meanings, are useful when communicating internationally, and are more easily understood than abstract images [9,15,21,32,33]. However, they cannot accurately represent abstract concepts, such as emotions or situations, which are dependent on cultural contexts.

Opaque symbols on the other hand do not have a clear relationship with their referents [20]. Although they can represent complex and abstract concepts, they may not be immediately recognizable and must be learned beforehand [31,32,35].

Symbols can also be separated into the monosemic, polysemic, and pansemic image categories [6]. Monosemic images only have 1 meaning, polysemic images have 2 or more meanings, and pansemic images have many meanings. Typically, abstract images are pansemic; however, monosemic connotations are preferable when developing a pictogram to communicate information about hazardous substances [6].

Pictograms based on existing systems have more transparent connotations [35,38]. Thus, as a person becomes more familiar with a certain type of visual language, they become more apt at interpreting different pictograms, provided that the pictograms follow the same visual synthesis [35,38].

In this study, requirements were taken from the designs of hazard and health-related pictograms for the development of the pictograms that were used to facilitate privacy agreement understanding. The requirements are as follows: (1) making the pictograms with users; (2) testing the pictograms with users; (3) developing the pictograms by using an iterative method; (4) building upon pictograms from existing systems; (5) using pictures with labels, keywords, or short texts; (6) using color; and (7) making the pictograms culturally relevant.

The objectives of this study were to develop a set of pictograms that represent the top 10 privacy concerns, assess their impact on users’ understanding, and encourage users to engage with privacy agreement content. The hypothesis is that with the incorporation of visual assistance, the users will find reading privacy agreements easier and less frustrating.

## Methods

### Design Method for the Development of Pictograms

This research was part of a larger project that focused on trust and privacy agreements. The larger project was divided into the following three phases: (1) identifying the top 10 privacy concerns, (2) exploring the use of pictograms for privacy agreements, and (3) assessing the effectiveness of the new privacy agreement layout. This research focused on phases 2 and 3, using the results gathered from phase 1.

**Figure 1:**
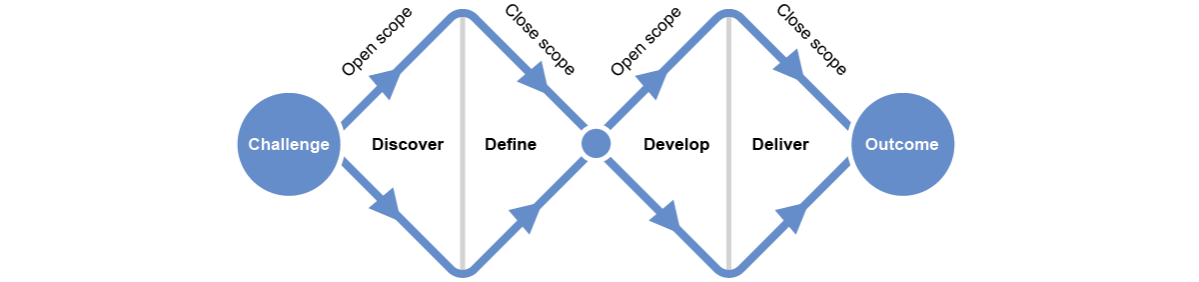
Double Diamond design method.

The methodology that was used to develop the pictograms was based on the Double Diamond design methodology (Figure 1). It was chosen for its iterative nature and the many points of contact between the designers and users.

The methods were divided into the following four phases: the *Discover*, *Define*, *Develop*, and *Deliver* phases. During the *Discover* phase, the research scope was expanded to understand the users’ needs and opinions. The *Define* phase was used to narrow the scope and analyze the collected data to identify trends, themes, and patterns. In the *Develop* phase, techniques such as brainstorming, sketching, and graphic recording were used to further develop previously identified ideas. The scope was closed a final time in the *Deliver* phase, during which a solution was prototyped and tested by users.

In this study, contact with users only took place during the *Discover*, *Develop*, and *Deliver* phases. A total of 9 participants were included in a visualization exercise for the *Discover* phase. They were asked to sketch their ideas for the visual representations of the top 10 privacy concerns on paper and to briefly explain what they were thinking when they made these representations. These visualizations were analyzed for trends and patterns during the *Define* phase. Afterward, based on these patterns, the pictograms were constructed during the *Develop* phase. Lastly, in the *Deliver* phase, the pictograms and a version of a privacy agreement that implemented them were validated by a group of users through a questionnaire.

### Evaluation of Overall Understanding and User Frustration

An evaluation was conducted to test whether the addition of the pictograms made reading privacy agreements more efficient and less frustrating for users. For this purpose, a questionnaire was developed along with 2 versions of the privacy agreement. The control group (31 participants) received the traditional version of the privacy agreement while the experimental group received the version of the privacy agreement that included the pictograms (29 participants).

The survey was closed and distributed through Amazon Mechanical Turk—a website that allows people to fill out surveys for a small monetary gain. The administration of the survey was performed via Amazon Mechanical Turk, and security for the survey and the assurance that there were no duplicate responses were provided by the website. All questions were multiple-choice questions, and if there was a question that was not properly filled, the data for that whole entry were discarded, which happened only once.

In total, 62 people started the survey and 57 people completed it. The target population was people who had some understanding of technology, and the sample was a convenience sample. The data were collected during the first week of September 2019.

Both groups were quizzed on the content of their version of the privacy agreement and were later asked to rate their perceived level of frustration when looking for the answers. Participants were then asked for suggestions about changes to the privacy agreement and the pictograms.

The 4-part questionnaire was developed by using Qualtrics (Qualtrics International Inc)—a web-based tool—and beta tested via a pilot study to assess its feasibility. The first part asked demographic questions about participants’ age, sex, ethnicity, occupation, education, country of residence, and region. We used the second part to compare the performance of the control group to that of the experimental group for part 3. In the second part, the control group was given the traditional version of the privacy agreement, whereas the experimental group was given the version of the privacy agreement with a group of pictograms that summarized its content, which appeared before the written section. Both versions of the privacy agreement can be viewed in Multimedia Appendix 1.

Participants were then asked to answer 5 questions that quizzed them on the content of the privacy agreement that they had received. For both groups, all questions were about the information represented by the pictograms.

The questions were as follows, and each question was given its own page on the survey:

- Question 1: “Is your information being collected?”
- Question 2: “Can you opt out of some services?”
- Question 3: “Will your data be identifiable when shared?”
- Question 4: “Is your location being collected?”
- Question 5: “Can third parties have access to your data?”

Each participant’s response was timed to assess how quickly participants could find the correct answers based on the information presented in their version of the privacy agreement. Time data were compared between the control group and the intervention group.

The third part of the questionnaire asked participants to rate their frustration levels while answering part 2, their level of concern, and their previous knowledge about data privacy. In total, there were 9 pages in the survey, which included the option to return to the previous pages before the end of the survey.

### Evaluation of Pictogram Efficiency

In the fourth part of the survey, participants were asked to take an estimated comprehension of symbols test [39] to measure how comprehensive the pictograms were for public use and to determine what further revisions would be required.

Each pictogram was presented individually, coupled with a description of what it was supposed to represent, without a legend. Participants were asked to rate the percentage of the population that they thought would be able to understand the pictogram and the description.

**Figure 2:**
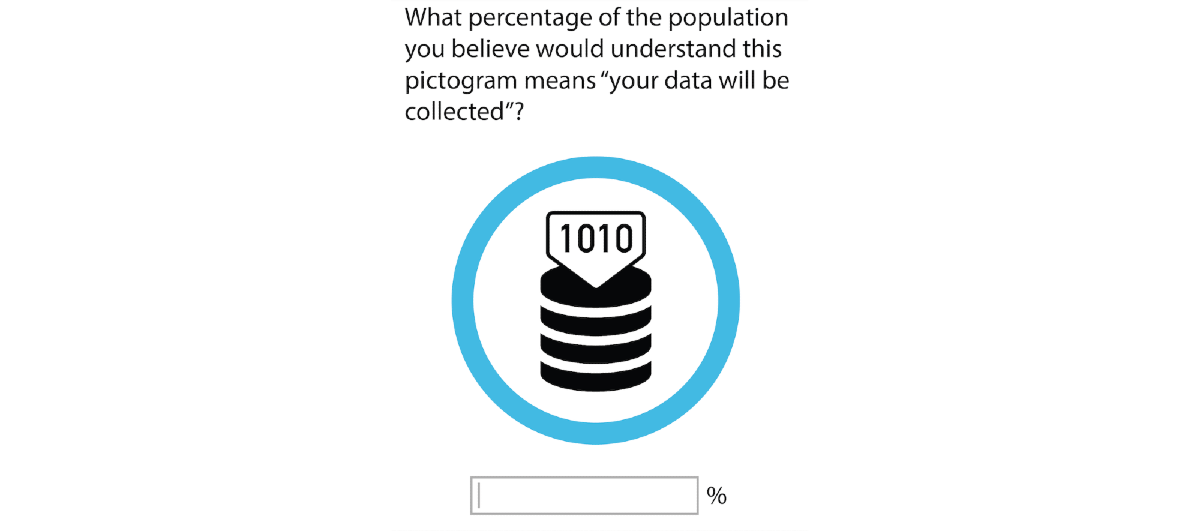
Example of how the estimated comprehension of information symbols test was applied.

For cases with an estimated comprehension level of <47%, the symbol was considered a failure. On the other hand, an estimated comprehension level of >87% was deemed appropriate. An estimated comprehension level of between 47% and 87% implied false negatives and false positives. For such cases, the symbol would have to be tested again by using a classic comprehension test [39]. An example of how the fourth part of the questionnaire looked can be found in Figure 2.

### Ethics Approval

The survey was cleared by the University of Waterloo ethics board (application number: 4060 Privacy Agreement for Sharing Health Data), and was registered with Clinical number NCT05631210. The survey was voluntary, and participants could stop participating at any moment. At the start of the questionnaire, the participants were told about the purpose of the study, its length, the possible risks, and the benefits of taking the survey. They were then asked for informed consent. The only personalized information collected was employment status, sex, age, ethnicity, and the places where participants lived.

## Results

### Participants

A total of 57 participants were recruited by using Amazon Mechanical Turk; 28 completed the questionnaire with the privacy agreement that implemented the pictograms, and 29 completed the one with the original, imageless privacy agreement.

The sample consisted of 22 female participants and 35 male participants who resided in the United States (n=18), Canada (n=19), or Europe (n=18). The Europeans were from the United Kingdom (n=6), the Netherlands (n=2), Italy (n=3), Germany (n=2), France (n=3), Spain (n=1), and Estonia (n=1). The distribution of ethnicities was White (n=49), Black (n=3), Chinese (n=2), South Asian (n=1), Southeast Asian (n=1), and Filipino (n=1). The rest of the participants’ demographics and occupations are described in Multimedia Appendices 2 and 3.

Different levels of interest in data privacy were reported; 21 participants reported high levels of concern about data privacy, 20 expressed moderate concern, 10 had low concerns, and 6 were neutral. Further, 35 participants thought that data privacy was highly important, 13 considered it to be moderately important, 4 believed it had little importance, and 5 were neutral.

**Figure 3:**
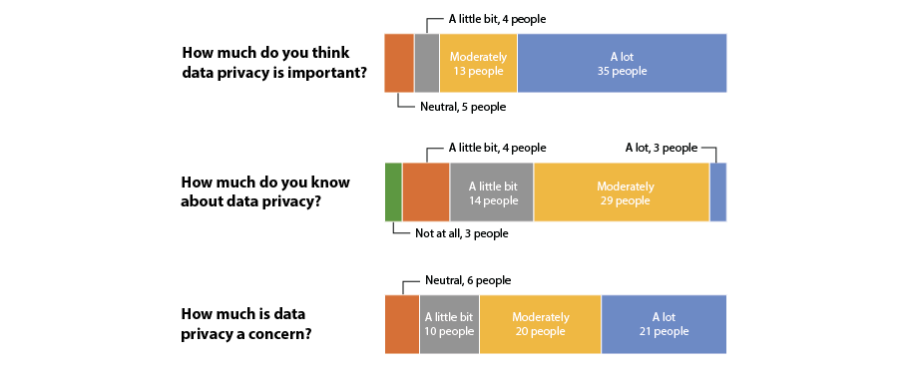
Concerns and knowledge about data privacy reported by participants.

With regard to previous knowledge about data privacy, 3 participants claimed to be highly knowledgeable, 29 claimed to have moderate knowledge, 14 claimed that they had little knowledge, 8 felt neutral about their knowledge, and 3 claimed to have no knowledge. These variations in concerns and knowledge levels can be reviewed in Figure 3.

### Development of Pictograms

#### Overview of Pictogram Development

A representation exercise was conducted during a workshop with 9 members of the Ubiquitous Health Technology Lab at the University of Waterloo. The participants were informed of the top 10 privacy concerns one at a time and were asked to create visual representations that they felt would accurately embody each concern.

**Figure 4:**
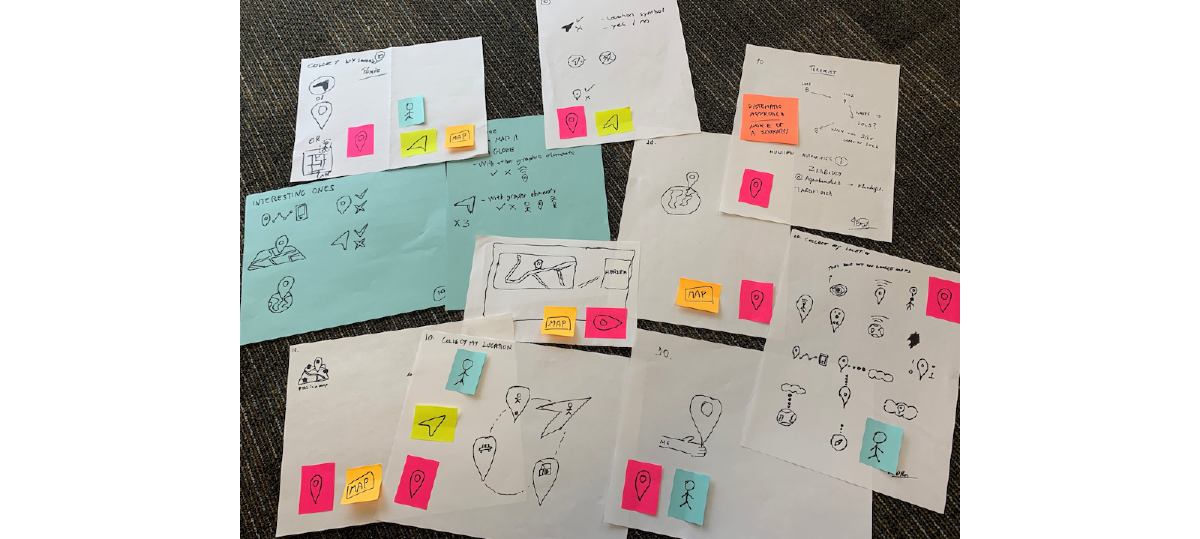
Representations collected during the workshop to represent the concept of “is my location being collected?”

In total, 90 pages of visualizations with varying degrees of representational content were collected to represent 10 privacy concerns. Figure 4 shows an example of the representations collected for one of the privacy concerns—“Is my location being collected?” Each white page belonged to a single participant, and the colored stickers represented the most common elements across the sample. Finally, the blue papers summarized the most used elements within the sample for a given privacy concern.

A content analysis, which followed the *Define* phase of the Double Diamond design method, was completed to organize and identify the patterns and trends within the representation ideas. The most used elements throughout the visualizations were (1) arrows or the notion of direction (n=56), (2) representations of the self (n=41), (3) clouds (n=23), and (4) binary code (n=17).

The first set of pictograms was developed by using the results from the visualization exercise, and they can be found in Multimedia Appendix 4. The pictograms were based on material design icons, in accordance with one of the guidelines sourced from the literature [40]. There were 3 iterations of pictograms, and with each iteration, implementation feedback was solicited from the team of design professionals.

We used one-on-one interviews to acquire feedback, during which the context was explained to the participants. They were given a single sheet of paper with all of the pictograms printed on it. After the interviews, the researcher asked each participant to explain what they thought each pictogram represented. Afterward, the researcher told the participant what the intended meanings were and asked them to propose changes that they believed would improve comprehension.

**Figure 5:**
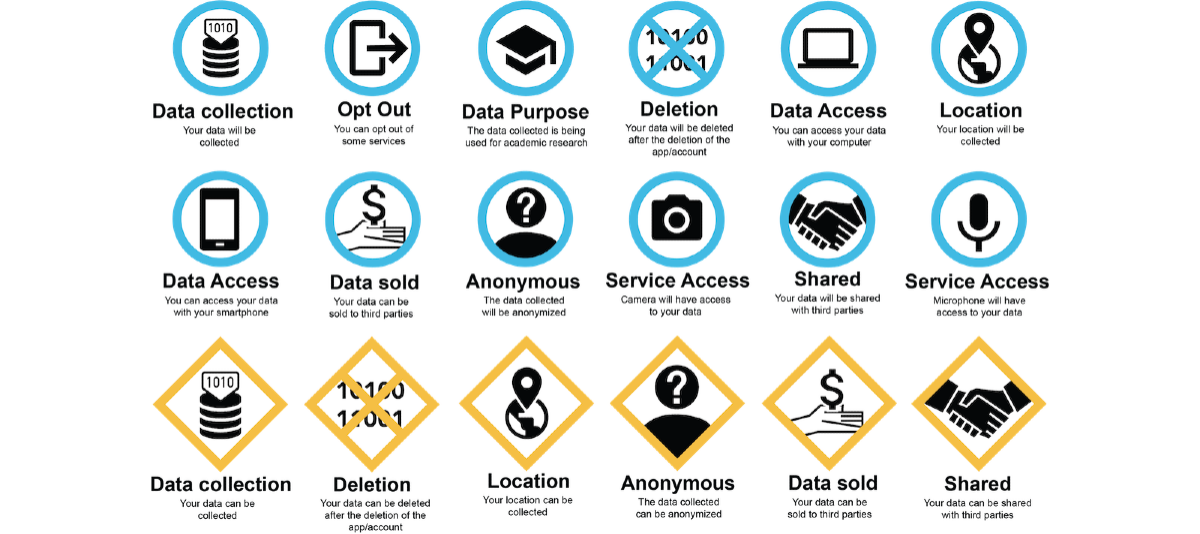
Final set of pictograms developed.

The responses were audio-recorded and then analyzed to detect whether the participants had guessed the meaning of a pictogram correctly. After the first interview, a second set of pictograms was developed, and this can be found in Multimedia Appendix 5. This set passed through the same interview process as the previous set to create the final set (Figure 5).

The final set of pictograms was divided into the following three categories: pictograms that addressed what the user could choose to do, pictograms that presented facts that could not be changed by the user, and pictograms that showed the user what the system permitted them to do.

#### User Possibility: What the User Can Choose

The first category of pictograms showed the user what options were available for them to choose. In this category, the pictograms were for “data being collected,” “data will be deleted after the deletion of the app/account,” “location is being collected,” “data collected is anonymized,” “data being sold,” and “data can be shared with third parties.”

These pictograms were designed differently from those in the other two categories. They had a yellow frame shaped like a square on one of its axes to mimic warning pictograms. This shape was chosen to attract more attention, since these pictograms showed the user what information they had control over.

#### User Impossibility: What the User Cannot Choose

The second category contained pictograms that presented characteristics of the system that the user had no control over. The pictograms in this category were counterparts to all of the pictograms in the *User Possibility* category, with additional pictograms for “microphone will have access to your data,” “camera will have access to your data,” and “your data will be collected for academic purposes.” These pictograms had the same core black and white symbol but had a circular blue frame.

#### System Characteristics: What the System Lets the User Do

This category of pictograms showed what the system allowed the user to do. The pictograms in this category were “opt-out,” “data access through your computer,” and “data access through your phone.” These pictograms had the same blue circular frame as those in the *User Impossibility* category.

### Overall Understanding of the Privacy Agreements With Pictograms

**Figure 6:**
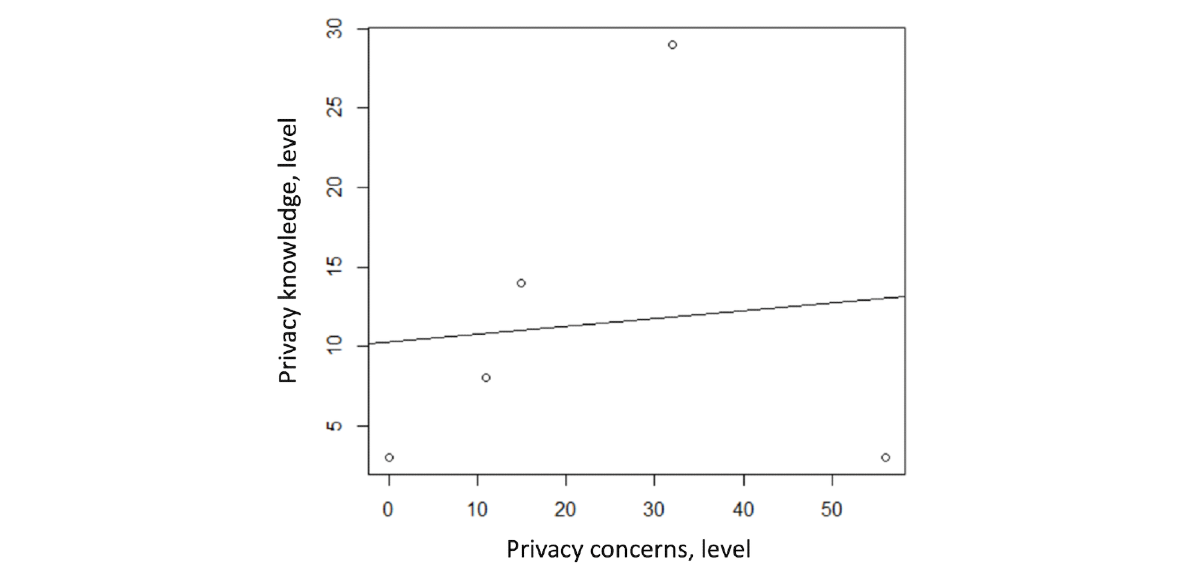
Relationship between privacy concerns and knowledge.

A 2-tailed Pearson correlation test was performed to assess if there was a relationship between privacy concerns and knowledge. There was a slightly positive correlation between privacy concerns and knowledge, but it was not significant (*r=*0.10, df=98; *P*=.87), as shown in Figure 6.

Introducing pictograms improved the overall understanding of privacy agreements by 13%. The original layout resulted in 106 right answers, 28 wrong answers, and 11 people who did not know the answer. The layout with the pictograms resulted in 121 right answers, 8 wrong answers, 3 people who did not want to read the privacy agreement, and 8 people who did not know the answer. Participants in the experimental group chose the correct answer 13% more often than the control group but chose the “didn’t know the answer” option 2% less often than the control group. However, they also chose the “didn’t want to read” option 2% of the time, while no one selected the same option in the control group.

Fisher exact tests (Table 1) were performed for each answer type to determine differences in levels of understanding between the two groups. This study found that participants’ understanding was significantly associated with the privacy agreement layout with which they were presented (*P*=.001).

Although the increased accuracy of answers that was observed with the addition of the pictograms was not significant (*P*=.008), this improvement demonstrates that participants still had a better understanding of the privacy agreement content if images were presented alongside text.

**Table 1:** Fisher exact test (*P*=.001) for understanding.

Answer type	Understanding of privacy agreement with pictograms, SE	Understanding of original privacy agreement, SE	*P* value
Right answer	0.03	0.03	.008
Wrong answer	0.01	0.02	<.001
Did not want to read privacy agreement	0.01	0	.12
Did not know answer	0.01	0.01	.64

### Time Spent Reading the Privacy Agreements

The amount of time spent reading the privacy agreement and answering the five questions decreased for all questions except for the first one. Moreover, 1-way Mann-Whitney *U* tests (Table 2) were conducted for each question to investigate whether the decreases in time between the two privacy agreement versions were significant. The only significant decreases in time were observed for questions 2 (*P*<.001) and 4 (*P*=.004).

The average time for answering all 5 questions decreased by 57.33 seconds with the addition of the pictograms, suggesting that their addition assisted users in finding the correct answers faster. A potential reason for this could be that users did not read the original privacy agreement and only used the summarized information that the pictograms displayed, or the pictograms helped users understand the presented information better. However, considering that the overall understanding of the privacy agreement improved with the addition of the pictograms, it is more likely that they helped users gain insight into privacy agreements while also decreasing the time spent searching for specific information.

**Table 2:** The 1-way Mann-Whitney U test for the time spent reading the privacy agreement.

Question		Privacy agreement with pictograms	Original privacy agreement	Wilcoxon test statistic	*P* value
**Question 1**				481.00	.89
	Time spent (seconds), median	48.66	38.17		
	Time spent (seconds), mean (SD; SE)	87.40 (112.13; 21.19)	51.53 (51.41; 9.55)		
**Question 2**				193.00	<.001
	Time spent (seconds), median	8.80	39.44		
	Time spent (seconds), mean (SD; SE)	18.08 (23.14; 4.37)	61.48 (65.59; 12.18)		
**Question 3**				330.00	.12
	Time spent (seconds), median	8.99	30.90		
	Time spent (seconds), mean (SD; SE)	22.81 (34.49; 6.52)	51.94 (84.35; 15.66)		
**Question 4**				239.00	.004
	Time spent (seconds), median	8.40	16.78		
	Time spent (seconds), mean (SD; SE)	11.34 (11.70; 2.21)	31.52 (42.87; 7.96)		
**Question 5**				366.00	.27
	Time spent (seconds), median	6.58	5.46		
	Time spent (seconds), mean (SD; SE)	12.71 (24.84; 4.69)	13.19 (14.64; 2.72)		

### Perceived Frustration While Reading the Privacy Agreements

Users in the experimental group reported experiencing less frustration compared to the control group. There was 9% less perceived frustration in the experimental group. For the original layout, 24 people were neutral in terms of frustration, 23 reported being a little frustrated, 22 were frustrated, 15 were very frustrated, and 3 were extremely frustrated. For the layout with the pictograms, 31 participants were neutral, 18 were a little frustrated, 13 reported being frustrated, 16 were very frustrated, and 6 were extremely frustrated. Average levels of frustration (“a little bit frustrated” and “frustrated”) decreased by 14% with the addition of the pictograms. However, high levels of frustration (“very frustrated” and “extremely frustrated”) increased by 5% in the experimental group when compared to those in the control group.

**Figure 7:**
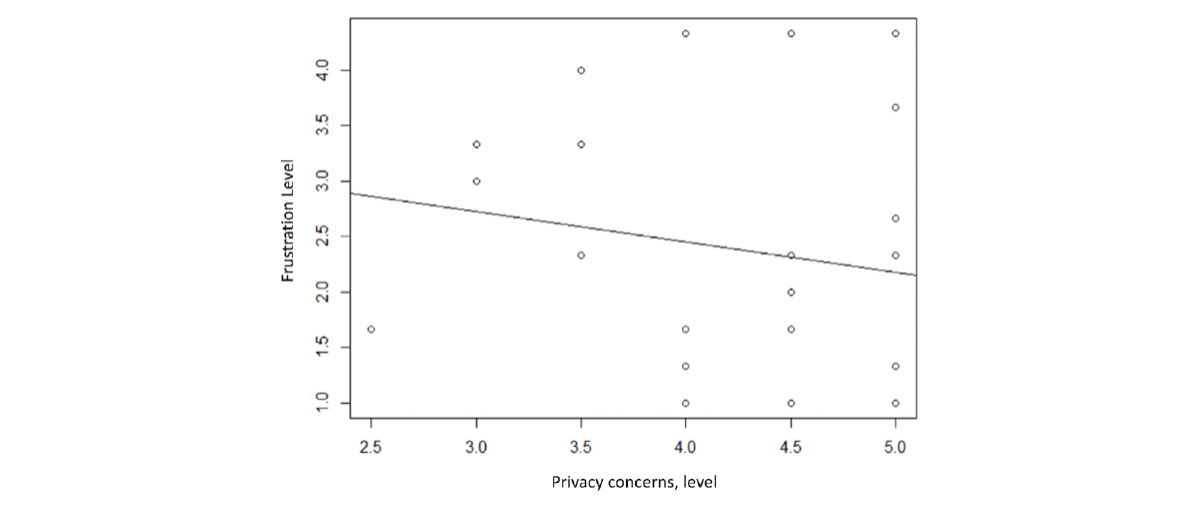
Relationship between privacy concerns and frustration (pictograms).

A chi-square test was performed to investigate if there were significant differences in perceived frustration levels between the two groups, and a 2-tailed Pearson correlation test was performed to assess if there was a relationship between the frustration and privacy concern levels. The chi-square test (Table 3) showed that overall frustration levels were not significantly different between the two layouts (*χ*^2^_4_=4.80; *P*=.31), and the 2-tailed Pearson correlation (Figure 7) test showed that there was a slight negative correlation between privacy concerns and frustration levels for the original version of the privacy agreement, though the negative correlation was not significant (*r*=–0.05, df: 98; *P*=.80).

**Table 3:** Chi-square test (*χ*^2^_4_=4.80; *P*=.31) and Fischer exact test for frustration.

Frustration level	Frustration with privacy agreement with pictograms, SE	Frustration with original privacy agreement, SE	*P* value
Neutral	0.03	0.03	.25
A little bit frustrated	0.02	0.03	.48
Frustrated	0.02	0.03	.13
Very frustrated	0.02	0.02	.84
Extremely frustrated	0.01	0.01	.32

### Estimated Comprehension of Information Symbols

None of the pictograms passed the estimated comprehension of information symbols test; 7 pictograms were discarded, as they had a score of less than 47%, and the remaining 5 pictograms were scored between 47% and 87% by participants and required further validation via a comprehension test (Table 2). The pictogram with the highest rating was “microphone is accessing your data,” with a 62.8% level of estimated comprehension, and the pictogram with the lowest rating was “your data is being collected.” A summary of the scores for all pictograms can be found in Multimedia Appendix 6.

Pictograms that relied on established material design icons and used transparent symbols based on suggestions made by Berthenet et al [9], Vaillancourt et al [16], Mok et al [33], Spinillo [35], and Mayer and Law [21] for designing pictograms had the best reception.

The ones that scored lower were the pictograms that used opaque symbols with pansemic meanings, which make pictograms harder to understand before they are incorporated into common knowledge [6,9,16,21,33]. A summary of which pictograms scored less than 47% and which ones scored between 47% and 87% can be found in Multimedia Appendix 7.

## Discussion

This research aimed to explore the use of pictograms for privacy agreements and assess the effectiveness of the new privacy agreement layout. Our findings suggest that the addition of pictograms improved the users’ experiences with understanding a privacy agreement when searching for information, even when suboptimal pictograms were provided. The decrease in the time taken to find the correct information and the self-reported decreased levels of frustration and confusion when engaging with the privacy agreements suggest a positive correlation between the addition of pictograms to privacy agreements and the perceived transparency of the documents’ contents.

To summarize, using images as an explanatory tool may improve the overall user experience when reading a privacy agreement and may even increase the understanding of the information being presented.

Even though the users considered none of the pictograms to be highly intuitive, the addition of the pictograms still helped users find the information about their data privacy settings, even when the pictograms’ meanings were less than transparent. We can assume that with the passage of time, these symbols will become integrated into common knowledge, will facilitate more interest in reading privacy agreements, and will result in such documents becoming more accessible to the general public, thereby fostering both trust and communication between users and the entities that collect their data.

## Appendix

Multimedia Appendix 1 Control group privacy agreement versus experimental group privacy agreement.

Multimedia Appendix 2 Demographics of participants.

Multimedia Appendix 3 Occupation of participants.

Multimedia Appendix 4 First set of pictograms developed.

Multimedia Appendix 5 Second set of pictograms developed.

Multimedia Appendix 6 Estimated comprehension of pictograms.

Multimedia Appendix 7 Pictograms to be discarded or further validated according to the estimated comprehension test.
