# Supplementary figures and images for: Exploring the Use of Pictograms in Privacy Agreements to Facilitate Communication Between Users and Data Collecting Entities: Randomized Controlled Trial
Source: JMIR Hum Factors. 2023 Jan 25;10:e34855. doi: 10.2196/34855 (PMC9947808; doi:10.2196/34855)

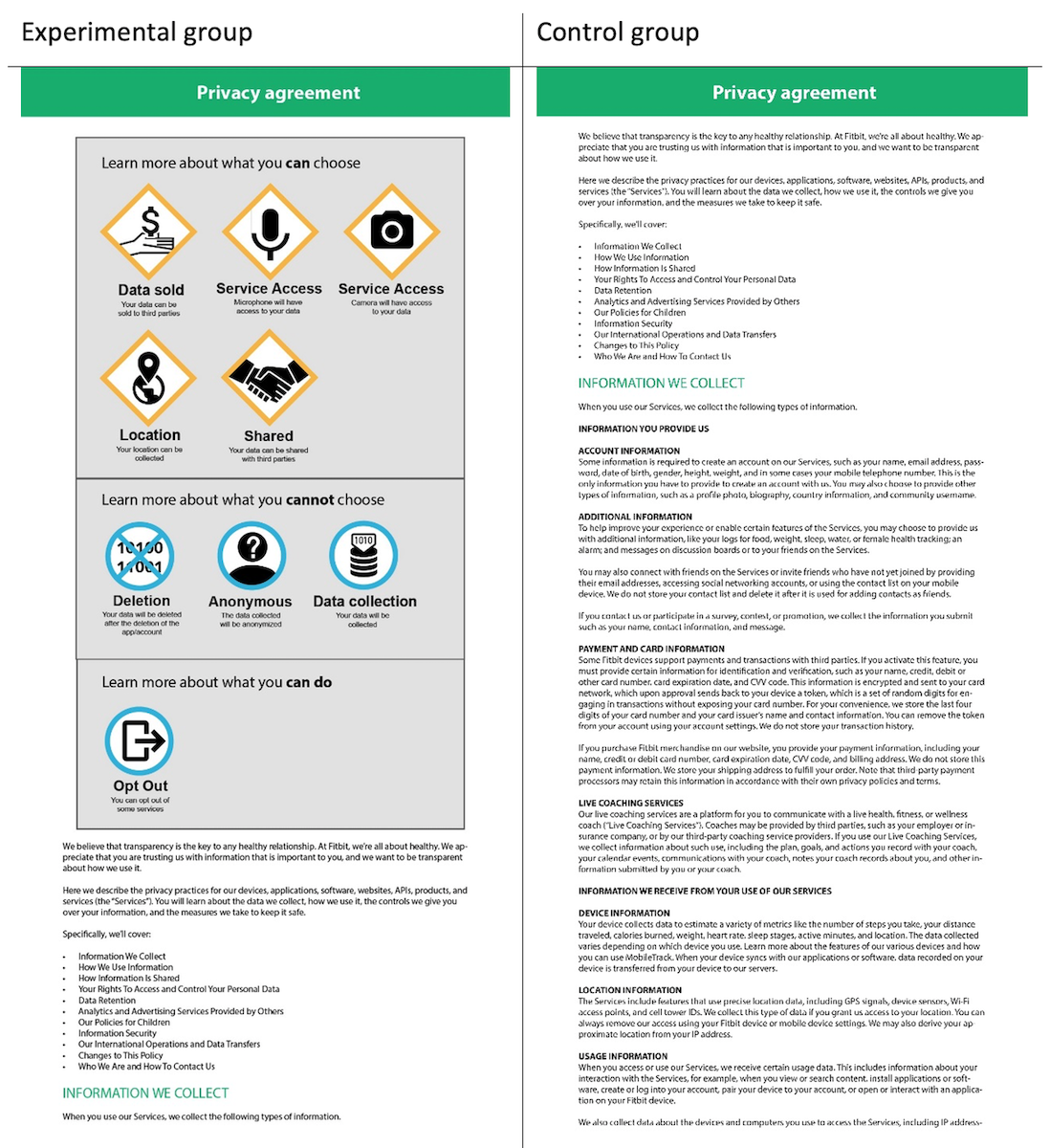

Supplement: Multimedia Appendix 1 [file humanfactors_v10i1e34855_app1.png]

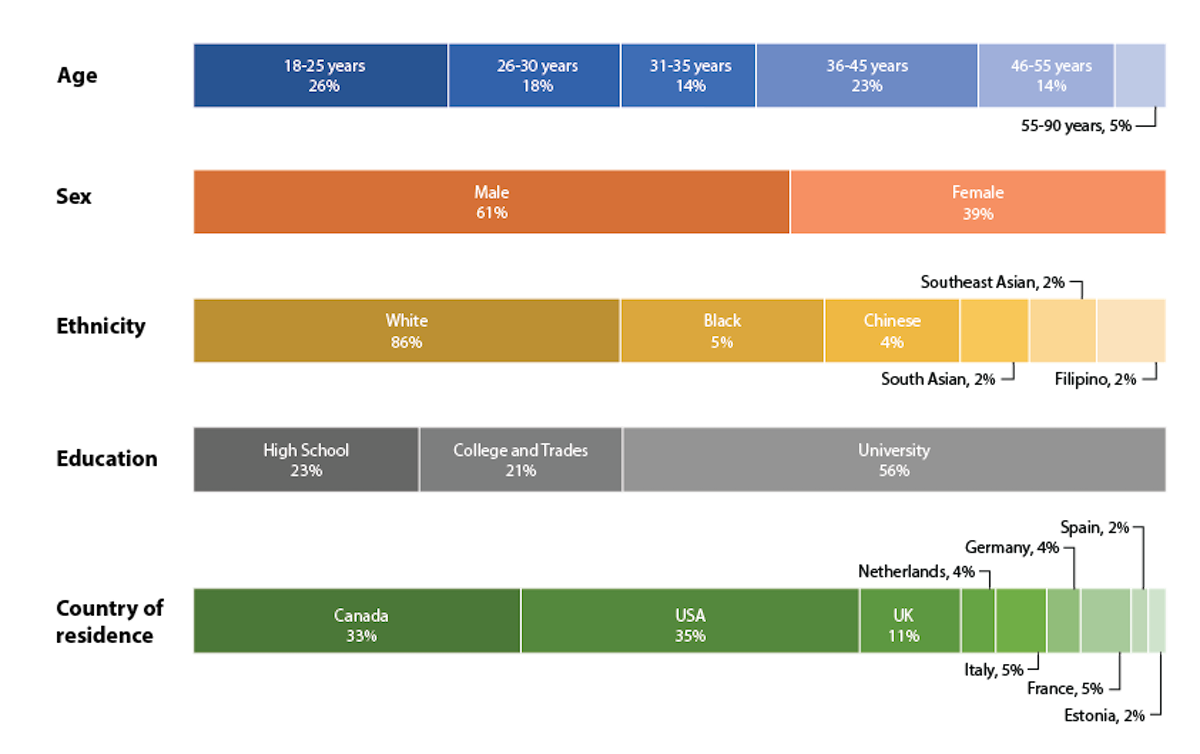

Supplement: Multimedia Appendix 2 [file humanfactors_v10i1e34855_app2.png]

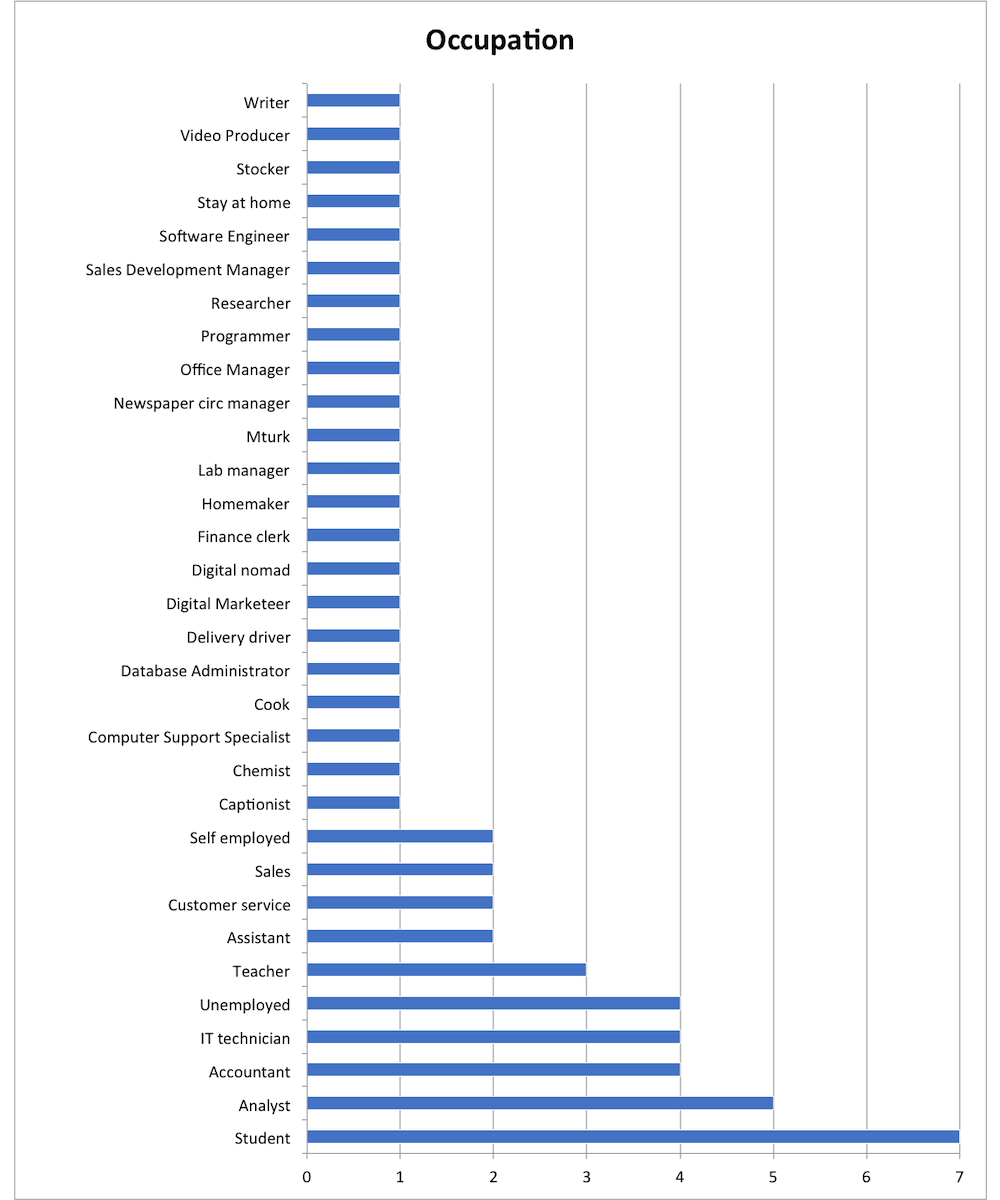

Supplement: Multimedia Appendix 3 [file humanfactors_v10i1e34855_app3.png]

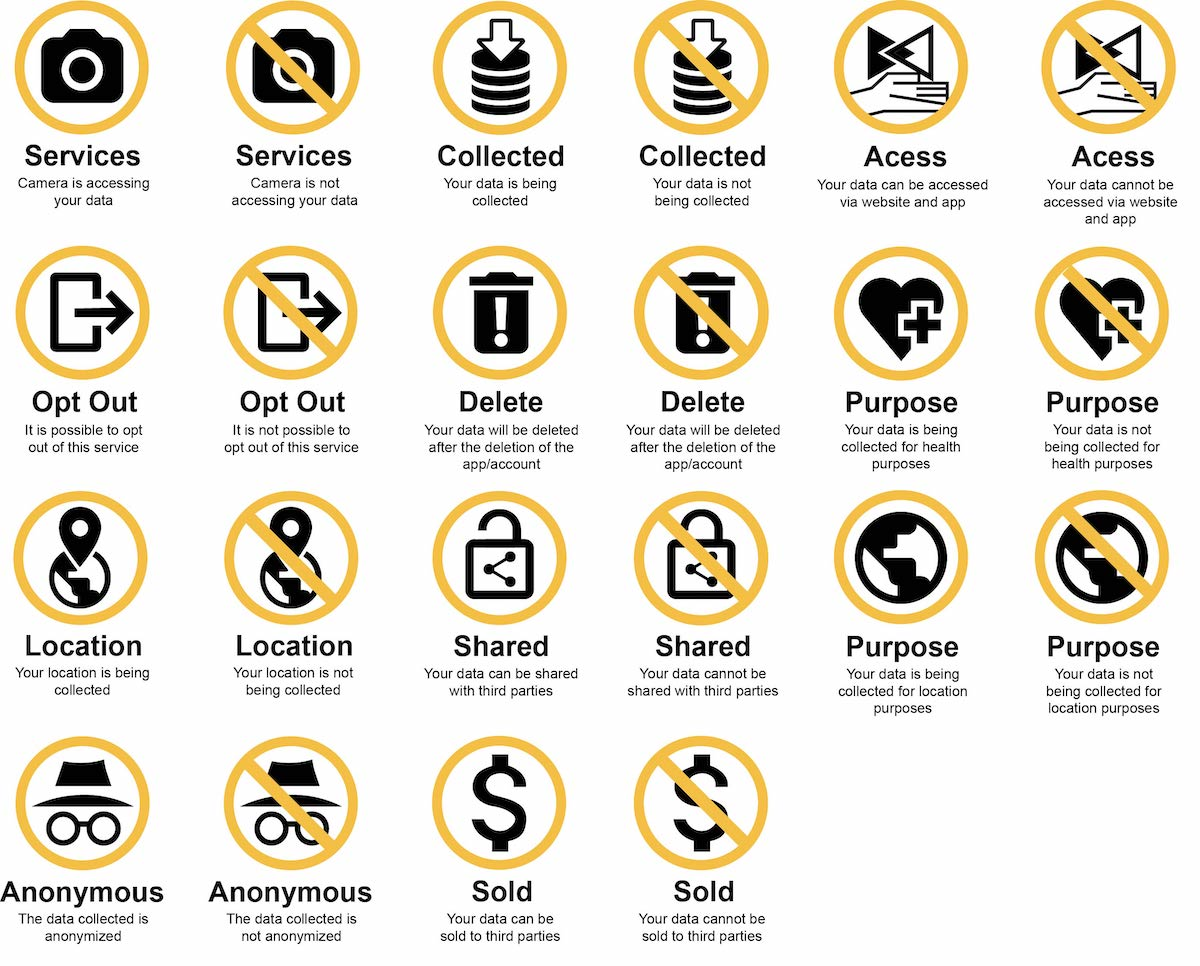

Supplement: Multimedia Appendix 4 [file humanfactors_v10i1e34855_app4.png]

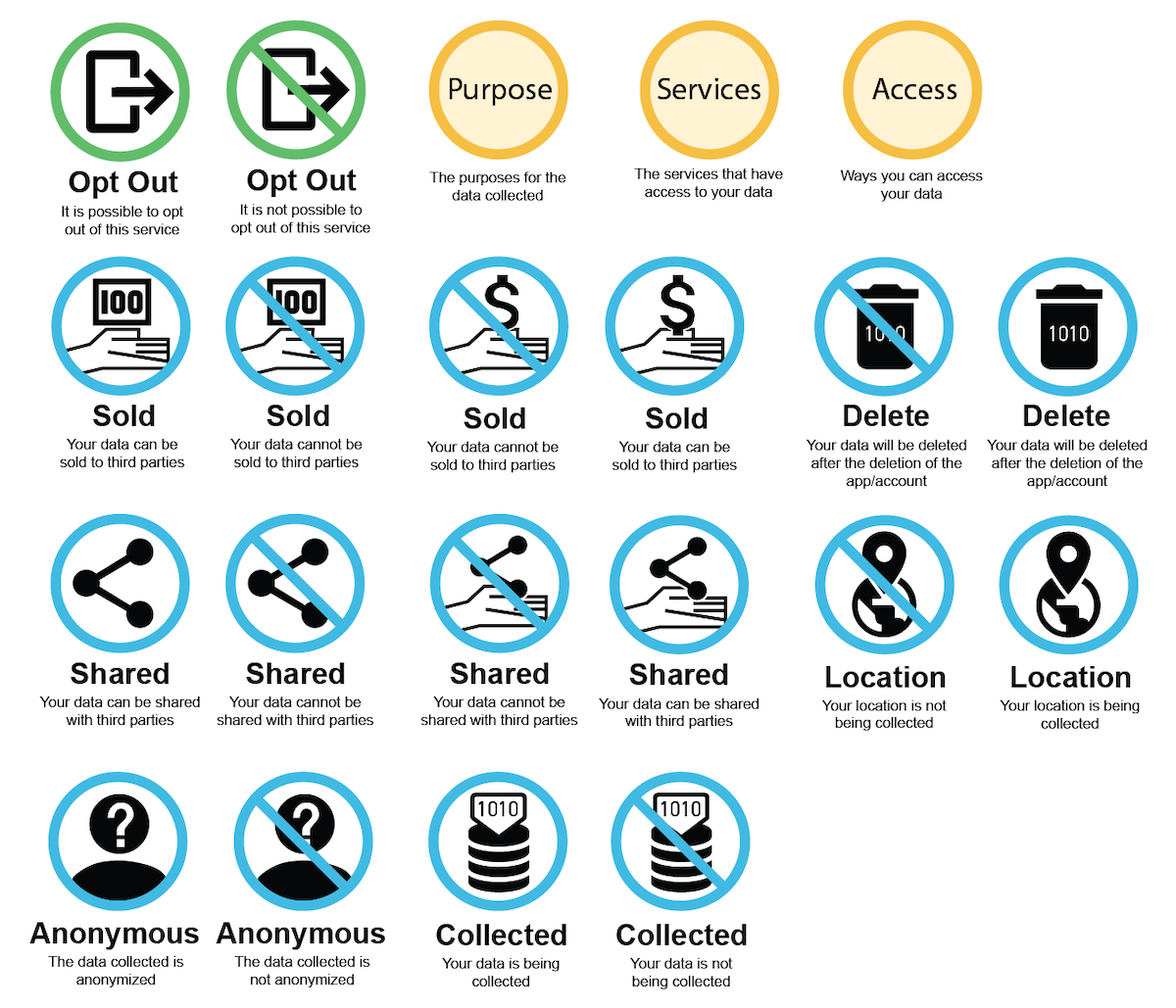

Supplement: Multimedia Appendix 5 [file humanfactors_v10i1e34855_app5.png]

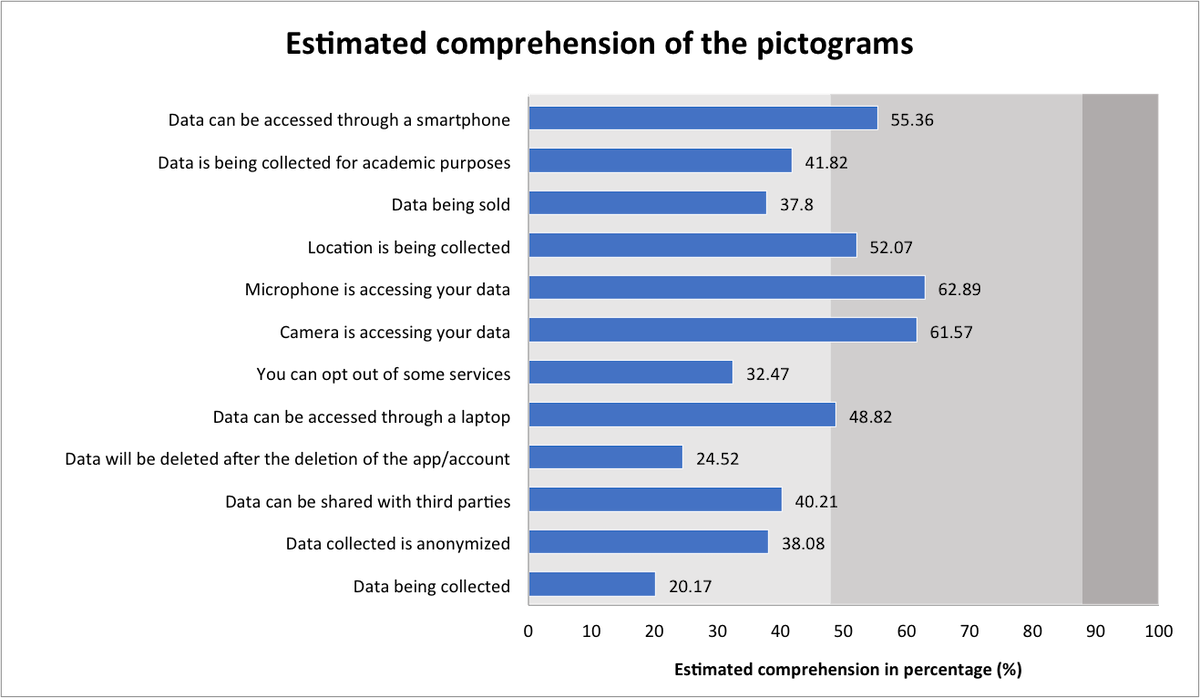

Supplement: Multimedia Appendix 6 [file humanfactors_v10i1e34855_app6.png]

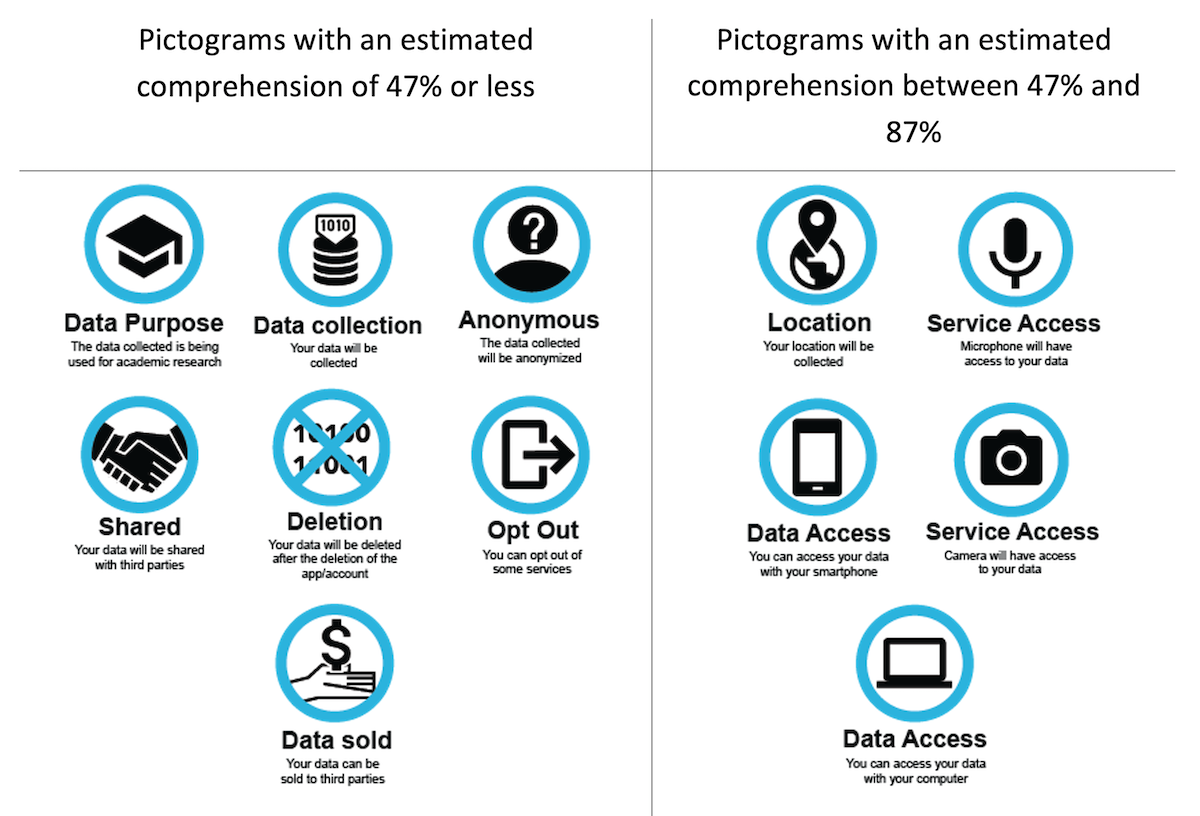

Supplement: Multimedia Appendix 7 [file humanfactors_v10i1e34855_app7.png]
